# Optical Characterization of Oligonucleotide DNA Influenced by Magnetic Fields

**DOI:** 10.3390/molecules181011797

**Published:** 2013-09-25

**Authors:** Seyedeh Maryam Banihashemian, Vengadesh Periasamy, Seyed Mohammad Hossein Mousa Kazemi Mohammadi, Richard Ritikos, Saadah Abdul Rahman

**Affiliations:** 1 Low Dimensional Materials Research Centre, Department of Physics, University of Malaya, Kuala Lumpur 50603, Malaysia; E-Mails: vengadeshp@um.edu.my (V.P.); richardritikos@gmail.com (R.R.); saadah@um.edu.my (S.A.R.); 2 Department of Basic Sciences, Payame Noor University, Tehran 19395-3697, Iran; E-Mail: smh_mousakazemi@pnu.ac.ir

**Keywords:** oligonucleotide DNA, magnetic field, refractive index, extinction coefficient

## Abstract

UV-VIS spectroscopic analysis of oligonucleotide DNA exposed to different magnetic fields was performed in order to investigate the relationship between DNA extinction coefficients and optical parameters according to magnetic-field strength. The results with the oligonucleotides adenine-thymine 100 mer (AT-100 DNA) and cytosine-guanine 100 mer (CG-100 DNA) indicate that the magnetic field influences DNA molar extinction coefficients and refractive indexes. The imaginary parts of the refractive index and molar extinction coefficients of the AT-100 and CG-100 DNA decreased after exposure to a magnetic field of 750 mT due to cleavage of the DNA oligonucleotides into smaller segments.

## 1. Introduction

The effects on biomolecules of exposure to a magnetic field have attracted a lot of attention in recent decades. Biomolecules can be easily manipulated by an external magnetic field; for instance, magnetic particles tagged to the biomolecule move by magnetic force and stretching, magnetic treatment and manipulation in tissue and blood [1,2,3,4,5]. In the broad biomaterials category, DNA strand manipulation by moderate intensity magnetic fields has been a topic a growing interest in biology and other scientific research. Immobilization of DNA strands on small chips, mechanical manipulation of DNA by magnetic fields in a magnetic tweezers device, and magnetic arrangement in liquid crystals are some interesting topics reported to date [4,6,7,8]. Magnetic sensors in metal-DNA-metal structures are another interesting recently reported application [9,10,11]. It is well known that the influence of a magnetic field on atoms and molecules has a significant effect on chemical reactions to create free radical intermediates possessing an odd number of electrons. Feasible electrical and medical applications of magnetic fields and DNA have opened up for us a new view focusing in this research on DNA manipulation and characterization. It should be pointed out here that DNA oligonucleotides with single strand nucleic acid sequences, due to their high sensitivity to detect small particles and virus (e.g., HIV), have scientific significance, prompting us to select this material for this research [12,13,14,15]. The subject of this work is the spectroscopic analysis of the DNA oligonucleotides adenine-thymine 100 mer (AT-100 DNA) and cytosine-guanine 100 mer (CG-100 DNA) influenced by magnetic fields. The magnetic field effect on DNA is investigated *in vitro* by optical methods for possible use in electronic devices and biosensor applications. The results show a significant effect on the DNA molar extinction coefficient and optical parameters after *in vitro* magnetic field exposure above 750 mT. This research concluded that these materials have the potential to be used in the biomedical field in diagnostic testing and also for bio-engineering research.

## 2. Results and Discussion

### 2.1. Results

The UV-VIS spectrum were measured to evaluate extinction coefficient and refractive indexes of the DNA oligonucleotides to the magnetic fields in controlled condition. The values of resistivity and temperature measured by a microchip electrode during the experiment showed increases of about 5 Ω and 2–3 °C, respectively, that were not significant.

#### 2.1.1. Extinction Coefficient

Determination on how strongly a DNA oligonucleotide absorbs light at a given wavelength enables calculation of its molar extinction coefficient, which is an intrinsic property of the material in the biological aspect. 

##### 2.1.1.1. Lambert and Beer Law

According to the Lambert and Beer (LB) law, the molar extinction coefficient for a DNA oligonucleotide can be calculated based on experimental UV-VIS spectroscopy data:

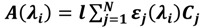
(1)

**Figure 1:**
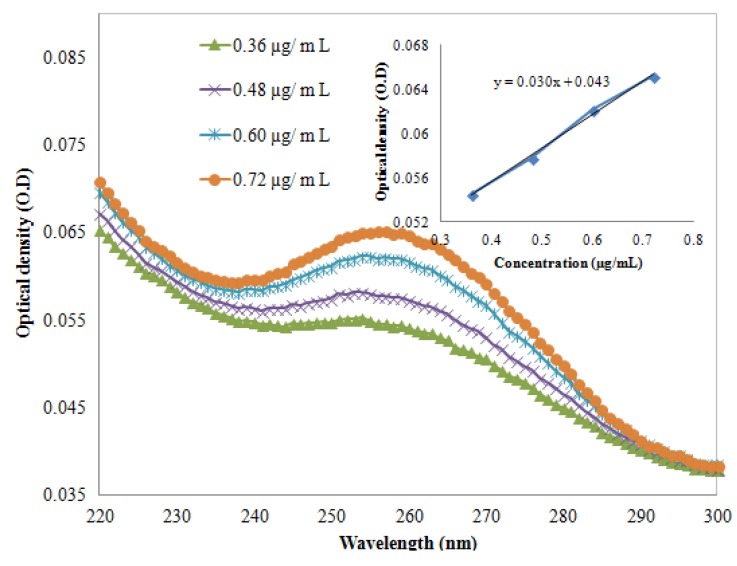
UV-VIS absorption via wavelengths at four concentrations. Inset shows the optical density *versus* concentration for AT-100 oligonucleotide DNA.

**Figure 2:**
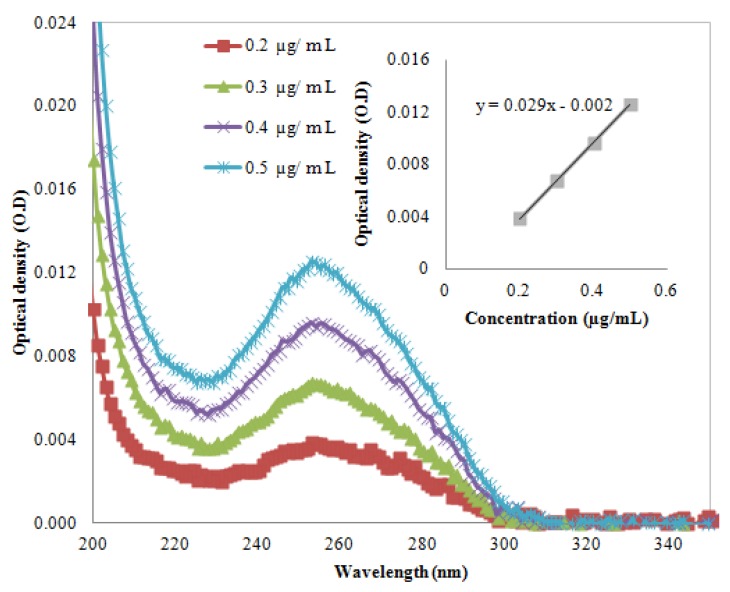
UV-VIS absorption in various wavelengths at four concentrations. Inset shows the optical density *versus* concentration for CG-100 oligonucleotide DNA.

Equation (1) shows the LB equation where 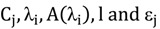 are the component concentrations, wavelength, absorbance, path length and molar extinction, respectively. The molar extinction coefficient was calculated after extracting the UV-Vis spectrum of the DNA (AT-100 and CG-100). Figure 1 and Figure 2 show UV-Vis spectra of two samples at a variety of concentrations. The insets in the figures show the relationship between optical density and concentration used to calculate the molar extinction coefficients, which for the single-strand oligonucleotides AT-100 and CG-100 are 927,352 and 896,789 L/mol·cm, respectively.

##### 2.1.1.2. Nearest-Neighbor

The molar extinction coefficient of a DNA oligonucleotide depends on the number of bases, which can theoretically be predicted from the sequence of the oligonucleotide. The molar extinction coefficient, is different for oligonucleotides with different sequences and would have to be ascertained theoretically by the nearest-neighbor and base composition methods [16,17]. In the nearest-neighbor method, the molar extinction coefficient of an oligonucleotide of length N can be given by the expression:

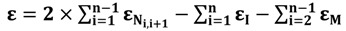
(2)
where 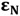 is the neighbor molar extinction coefficient of the neighboring nucleotides i, i + 1. The terms 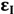 and 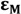 are the individual and modified molar extinction coefficients, respectively [18].

##### 2.1.1.3. Base Composition Method

In the base composition method, the molar extinction coefficients are the sum of extinction coefficients. The isolated nucleosides (A, C, G, and T) are multiplied [Equation (3)] by a factor of 0.9 to account for base stacking. A, C, G, and T are related by 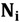, and 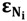 is the molar extinction coefficient for individual base pairs:

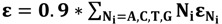
(3)

Experimental and theoretical calculations of 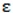 are shown in Table 1. The results for AT-100 and CG-100 were compared with each other; the experimental result was less than the theoretical investigation because of the double-strand’s hypochromicity effect.

**Table 1:** Theoretical and experimental result of 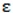 for the DNA oligonucleotides AT-100 mer and CG 100 mer (E-BC: Base composition method; E-NN: Nearest neighbor method; E-EX: Experimental result).

DNA Sample	E-BC (L/mol·cm)	E-NN (L/mol·cm)	E-EX (L/mol·cm)
AT (100 mer)	1,140,750 ± 5,703	1,082,685 ± 5,413	927,352 ± 4,636
CG (100 mer)	841,500 ± 4,207	846,016 ± 4,230	896,789 ± 4,483

**Figure 3:**
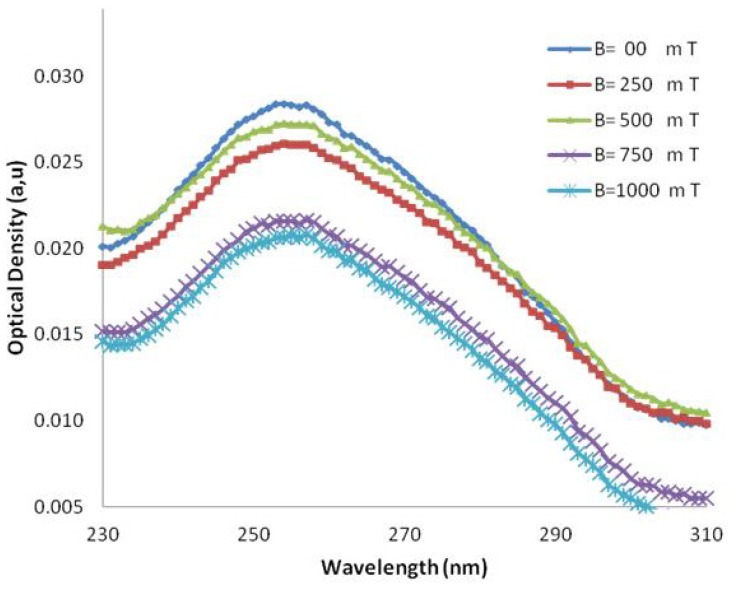
UV-VIS absorption in various wavelengths in several magnetic fields for CG-100 oligonucleotide DNA.

**Figure 4:**
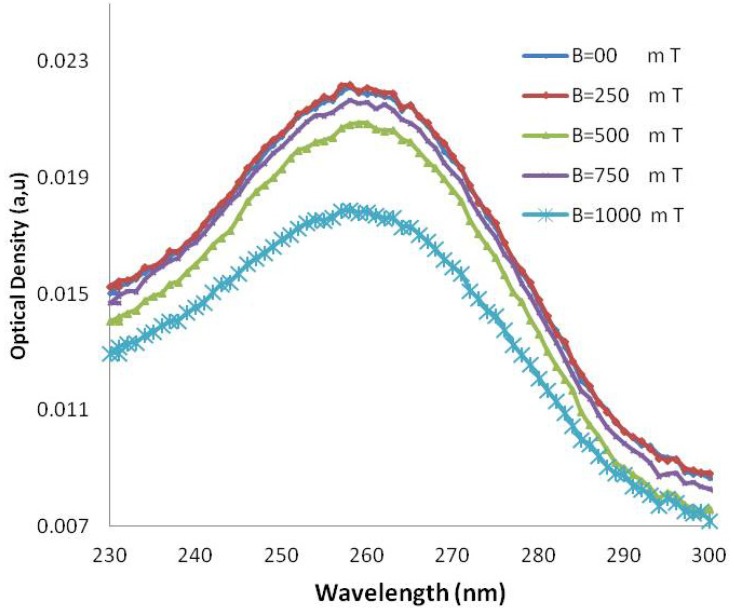
UV-VIS absorption in various wavelengths in several magnetic fields for AT-100 oligonucleotide DNA.

The UV-Vis spectra of DNA samples after exposure to several magnetic field strengths (250, 500, 750, 1,000 mT) are shown in Figure 3 and Figure 4, respectively. The *p*-values calculated for AT-100 and CG-100 by statistical regression analysis are 0.28 and 0.20, respectively, indicating a significant relationship between the molar extinction coefficient and the magnetic field strength.

**Figure 5:**
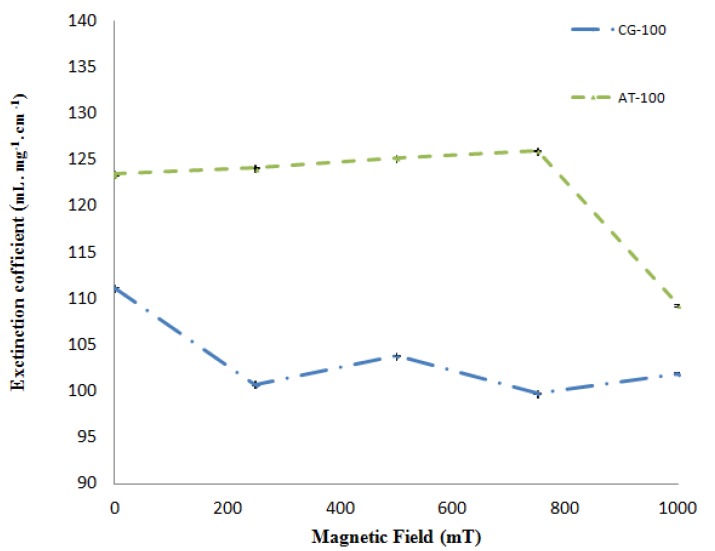
Extinction coefficients of the two DNA samples (AT-100 mer and CG-100 mer) after exposure to various magnetic field strengths.

Figure 5 depicts the molar extinction coefficients calculated for AT-100 and CG-100, respectively, *versus* the magnetic field. Based on these results, there was a decrease in the extinction coefficients after exposure to the magnetic field. 

The molar extinction coefficient of AT-100 is decreased significantly after exposure to a magnetic field above 750 mT, but there is an overall total decreasing trend in the molar extinction coefficients in CG-100. A segment of single-strand DNA with different charges at the two heads senses magnetic forces in the reverse direction, then the DNA divided into two or several parts with shorter length rather that the primary one. The shorter length DNA thus has a smaller molar extinction coefficient which decreases upon exposure to magnetic field due the small length stands.

#### 2.1.2. Refractive Index

The refractive index of tissues and biomolecules such as DNA is a fundamental parameter used in optical diagnosis tests and laser treatments. The Kramers-Kronig method is a crucial tool for studying the imaginary and real parts of the refractive index [19,20]. In the damped simple harmonic oscillator model, n and k are the real and imaginary part of the complex refractive index, respectively. The phase ϕ is related to the reflectance (R) by the dispersion equation, defined in the Kramers-Kronig analysis as follows:

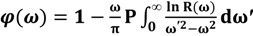
(4)
where P stands for the Cauchy principal value. The real and imaginary parts of the complex optical refractive index, n and k, respectively, are related to the reflectance amplitude and phase by the following equations [21,22]:

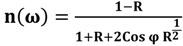
(5)

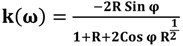
(6)

**Figure 6:**
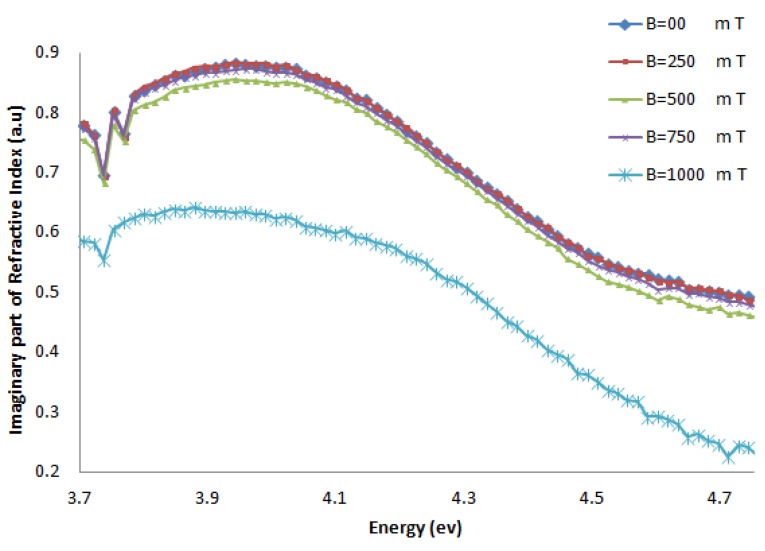
The imaginary part of the refractive index of AT-100 DNA exposed to magnetic fields.

**Figure 7:**
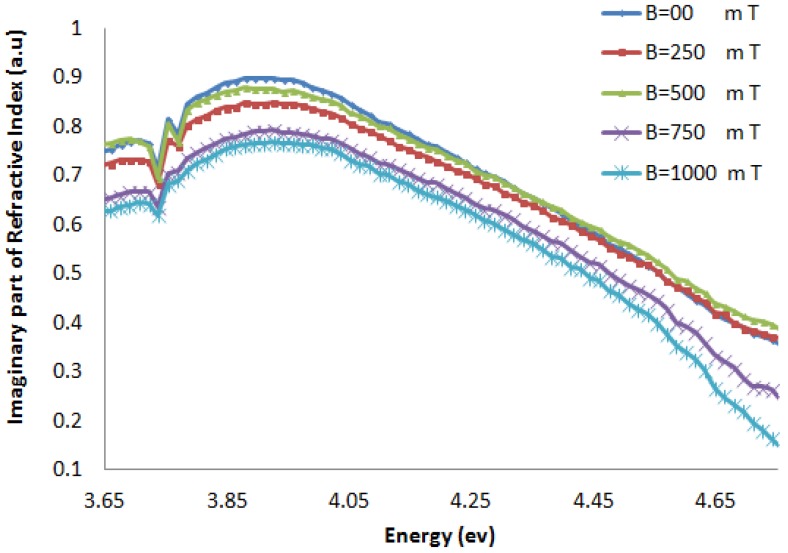
The imaginary part of the refractive index of CG-100 DNA exposed to magnetic fields.

**Figure 8:**
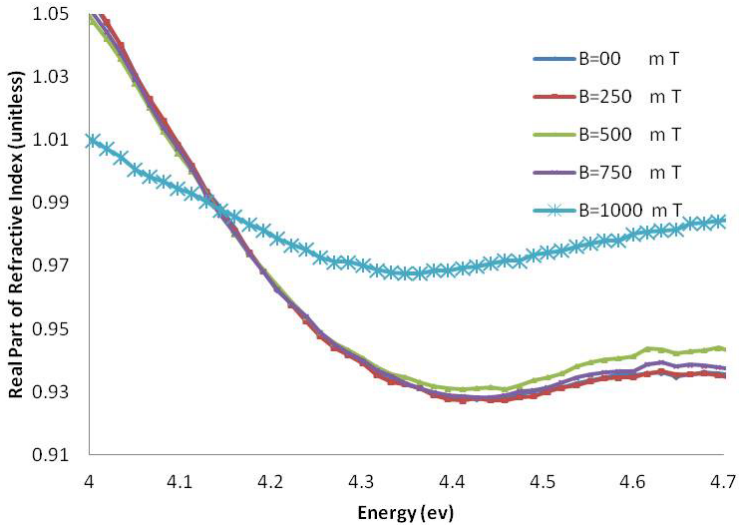
The real part of the refractive index of AT-100 DNA exposed to magnetic fields.

**Figure 9:**
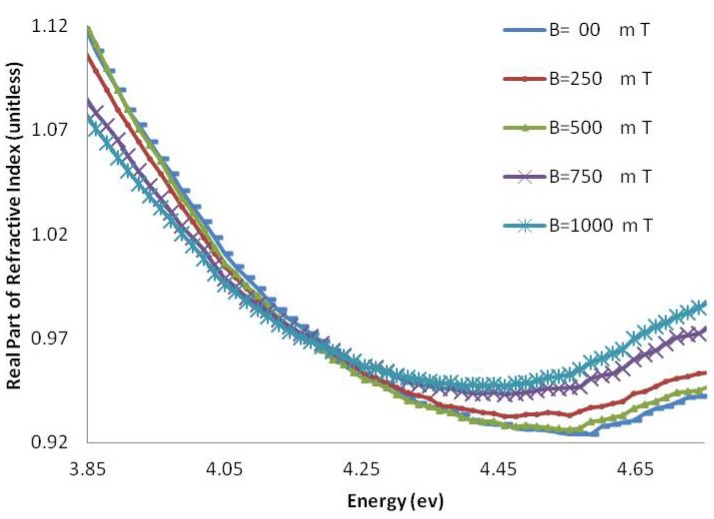
The real part of the refractive index of CG-100 DNA exposed to magnetic fields.

Figure 6 and Figure 7 show the imaginary part of the refractive index (k), and Figure 8 and Figure 9 indicate the real part of the refractive index (n) for AT-100 and CG-100, respectively; these values are derived from Equations (5) and (6).

The imaginary part of the refractive index of the AT-100 DNA and CG-100 DNA decreased after exposure to a magnetic field of 750 mT. This result confirms the biological aspect result in the molar extinction coefficient calculations. 

The real part of the refractive index of AT-100 and CG-100 changed after exposure to magnetic fields. A significant decrease in the refractive index was observed after exposure to magnetic fields of 1,000 mT. At energies less than the DNA absorbance peak position, there was a decrease in the real part of the refractive index and an increase in the region above.

### 2.2. Discussion

The real part of refractive index is related to the density of a liquid. In both samples, the density of DNA liquid samples increased at short wavelengths and conversely, are large wavelengths it decreased. In the other hand, the number of short length DNA oligos suspended in the liquid increased and the number of large wavelength ones decreased. This result indicates cleavage of the DNA oligos resulting in small segments.

The imaginary part of the refractive index is related to the extinction coefficient. The molar extinction coefficient and imaginary part of the refractive index decreased in both AT-100 and CG-100 after exposure to magnetic fields due to DNA cleavage that creates smaller length oligomers. Although, there are a some fluctuation in the refractive indexes and extinction coefficients because of reconnection of clipped DNA stands with the ionic head, the total trend shows a reduction in those parameters under the influence of the magnetic field. Generally, the number of bases in each DNA stand reduced after strand cleavage by the magnetic field and increased again by reconnection. According to Equations (1), (2) and (3), the shorter length DNA oligos have smaller extinction coefficients. 

## 3. Experimental

### 3.1. Materials

Two kinds of oligonucleotide DNA were used in this work. Adenine-thymine 100 mer (AT-100, molecular weight 30,818 and nanosize length 0.9 nm) and cytosine-guanine 100 mer (CG-100, molecular weight 30,859 g/mol and nanosize length 0.7 nm) were obtained by a small-scale PAGE purification of the synthesized oligonucleotides. The measured purity of samples, given by the ratio of the absorbance at 260 nm divided by the reading at 280 nm was 1.7–1.8, which is sufficient for DNA analysis. Immediately after suspending samples in 1 mM EDTA and 10 mM Tris at a controlled of pH 7.5 to 8.0, pre-incubation of the DNA oligonucleotides was carried out. They were centrifuged for 2 min at 6,000 rpm. After vortexing the tubes for 20 s DNA oligonucleotide (5 to 25 µL) was diluted with DI water based on the equation C_1_V_1_ + C_2_V_2_ = CV, where C and V are the concentration and volume, respectively, and exposed to a magnetic field. A p-type Si wafer (orientation <100>) possessing a resistivity of 1 to 10–20 Ω·cm (MEMC Electronic Materials, Saint Peters, MO, USA) was used as the substrate. The gold wire (Kurt J. Lesker Company, Clairton, PA, USA) used in the evaporation and magnetron sputtering technique had a purity of 99.999%. Other necessary chemicals (C_2_H_5_OH, DI water and acetone) and MicroChem’s SU-8 photoresist and developer were supplied by Sigma Aldrich (St. Louis, MO, USA) and were used without further purification.

### 3.2. Set up

**Figure 10:**
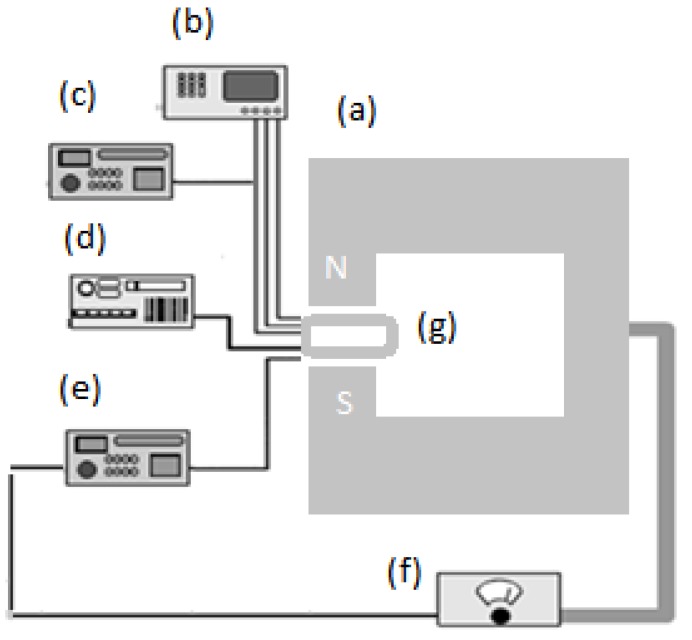
Prepared oligonucleotide DNA sample is placed inside magnetic field. (**a**) Electromagnet; (**b**) Thermometer; (**c**) Avometer; (**d**) Timer; (**e**) Gauss meter; (**f**) Electromagnet power supply; (**g**) DNA sample.

Figure 10 shows a schematic of the set up sequence. The magnetic field was applied inside two pairs of Helmholtz coils with variable distance. The variable homogenous magnetic field was provided by feeding current through the coils using a 2,500-watt DC power supply (4500 D, 3472-50 electromagnet, GMW, San Carlos, CA, USA).

The cell containing the oligonucleotide was located in the center of poles and held from 2 to 10 min at 250 °C. A thermometer, avometer, timer, and a Gauss meter to measure the flux density were utilized for controlling the physical parameters before and after exposure to the magnetic field (250, 500, 750, and 1,000 mT). UV-Vis spectroscopy was carried out using a PerkinElmer 750 instrument. The liquid temperature was controlled to calculate the resistivity during the magnetic field exposure. The temperature was increased by applying the magnetic field about 2–3 °C. All sample measurements were repeated at a fixed temperature (25 ± 2 °C) and resistivity (1,795 ± 5 Ω) to ensure controlled conditions with small chips fabricated by standard lithography processes. Data was analyzed using Matlab and Origin 8.0 software.

### 3.3. Chip Fabrication

To control resistivity and temperature during the experimental work, two gold electrodes with a separation of 40 μm were fabricated on a silicon wafer (microchip) based on a standard lithography process. The fabrication process is as follows: after substrate preparation and cleaning of the silicon wafers, the SU8 photoresist was deposited using spin coating with speed of about 1,500–2,000 rpm and one min. After this process, the UV light exposure through the designed mask via-lithography process was done and after 1 min of post exposure bake (PEB) at 95 °C followed by MicroChem’s SU-8 developer. One hundred nm gold deposition was achieved by thermal evaporation on the sample.

## 4. Conclusions

The effects of magnetic fields of varying strength on oligonucleotide DNA are investigated in this work. DNA 100-mer oligonucleotides of adenine-thymine (AT-100) and cytosine-guanine (CG-100) were exposed to magnetic fields of varying strength (250, 500, 750 and 1,000 mT). UV-Vis spectroscopy was used to determine the molar extinction coefficients and refractive indexes. The results indicate that the effect of exposing DNA oligonucleotides to magnetic fields is cleavage of oligomers. There are a significant decrease in the imaginary part of the refractive indexes and molar extinction coefficients in magnetic fields stronger than 750 mT.
